# Automatic topic identification of health-related messages in online health community using text classification

**DOI:** 10.1186/2193-1801-2-309

**Published:** 2013-07-10

**Authors:** Yingjie Lu

**Affiliations:** School of Economics and Management, Beijing University of Chemical Technology, Beijing, 100029 China

**Keywords:** Online health community, Text classification, Topic identification, Topic classification

## Abstract

To facilitate patient involvement in online health community and obtain informative support and emotional support they need, a topic identification approach was proposed in this paper for identifying automatically topics of the health-related messages in online health community, thus assisting patients in reaching the most relevant messages for their queries efficiently. Feature-based classification framework was presented for automatic topic identification in our study. We first collected the messages related to some predefined topics in a online health community. Then we combined three different types of features, n-gram-based features, domain-specific features and sentiment features to build four feature sets for health-related text representation. Finally, three different text classification techniques, C4.5, Naïve Bayes and SVM were adopted to evaluate our topic classification model. By comparing different feature sets and different classification techniques, we found that n-gram-based features, domain-specific features and sentiment features were all considered to be effective in distinguishing different types of health-related topics. In addition, feature reduction technique based on information gain was also effective to improve the topic classification performance. In terms of classification techniques, SVM outperformed C4.5 and Naïve Bayes significantly. The experimental results demonstrated that the proposed approach could identify the topics of online health-related messages efficiently.

## Background

Health issues are a primary concern for many people. Especially for the patients diagnosed with some serious diseases and their caregivers, they usually seek explanatory information about their disease or treatment. Traditionally, health professionals such as doctors are the primary source of medical information for patients. However, health professionals often fail to meet the patients' information needs fully. Many patients feel that doctors are too busy to answer their questions (Umefjord et al. 2003), or many doctors just tell their patients basic medical information but are not willing to take the time required to fully explain the details (Dickerson et al. 2006). This view was supported by the argument of Tyson (Tyson 2000), who suggested that there was a lack of attention to detail in the current doctor-patient relationship. Recently, the Internet is changing the way people experience life, including healthcare. Many studies on health communication have shown that patients are increasingly using the Internet for health information and support. A study of US-based cancer patients and their caregivers indicated that 80% of them were interested in health-related information on the Internet and 65% expressed an interest in online support groups (Monnier et al. 2002). Especially for those patients with chronic diseases, they would prefer to search online health information to be better informed about their illnesses (Bansil et al. 2006). In recent years, with the advent of some social media services, such as Wikipedia, FaceBook, online forums and medical Q&A, patients are more likely to obtain health information and share health experiences on these social media websites (Kinnane & Milne 2010). A recent survey showed that 34% of Internet users have read someone else’s commentary or experience about health issues in online news group, website, or blog, and 24% of them have consulted online reviews of particular drugs or medical treatments (Fox & Jones 2009). These health-related social media services enable patients to take a more active role in making decisions about their health through the use of social support and the ability to explore treatment options (Gerber & Eiser 2001). In addition, convenience and anonymity are also important reasons why patients turning to the Internet, where patients could obtain health-related knowledge easily and quickly, and meanwhile they are not embarrassed to ask health professionals online or communicate with online members about their conditions (Anderson et al. 2003).

Although different types of social media applications can be used to obtain health-related information, online health community is among the most popular social media services. In online health community, patients and their caregivers can share their experiences and exchange interesting information. The emotional support and encouragement offered by community members is also important for patients suffering serious illness and help them cope with their diseases significantly better than those who address serious diseases by themselves.

Different participants sought different types of information and support. Some patients undergoing treatment for their diseases would prefer to talk of more treatment knowledge. Some survivors, even if their diseases have been permanently cured, were still willing to share their experiences and advice on how they deal with life's daily challenges through online health community. Moreover, some patients with serious illness often posted some messages with their blessing only to get mutual emotional support. So different participants have their own purpose and they only showed interest in some specific topics. However, currently in online health community, the messages that belong to the same topic scattered across many different threads, which makes it difficult for the participants to summarize and search their interesting messages quickly. An alternative solution is to classify these messages into different categories according to their topics, thus enables participants to obtain a sense of what online health community is, quickly find the issues they concerned about, become involved in online health community more easily, thereby gaining valuable information for their health self-education, self-care and self-management. In addition, for the websites that provide health-related social media services, topic identification and categorization could assist the web designers and developers into optimizing the human-computer interface, providing personalized tools and functions such as topic tags to facilitate the users to search their interesting topics quickly. However, with the explosion of online messages, it is becoming impractical to classify the messages manually. To address this issue, we proposed a topic identification approach based on text classification technique for identifying automatically topics of the health-related messages in online health community.

Over the recent years, text classification techniques have been widely used for topic identification of online text. In terms of medical informatics, some previous studies primarily focused on medical literature and clinical narratives. Take the example of MEDLINE biomedical database. It is well known that the MEDLINE medical literature contains millions of references to biomedical journal articles, making it very hard for researchers to efficiently reach the most relevant documents by searching. To address this issue, many studies attempted to design text classification system for classifying MEDLINE documents automatically (Yetisgen-Yildiz & Pratt 2005). In addition, text classification techniques were also often applied into clinical narratives to distinguish different types of patients, so that personalized medical services could be provided for them. For example, researchers have proposed a topic modeling for matching patient education materials to patients clinical notes so that relevant education articles could be recommended automatically to the patients and help patients find appropriate patient educational materials (Kandula et al. 2011). However, these studies mainly focused on professionally written medical text, which differed significantly from user-generated medical text in online health community. Few studies have attempted to apply text classification techniques into topic identification of these user-generated medical text.

## Method

**Figure 1:**
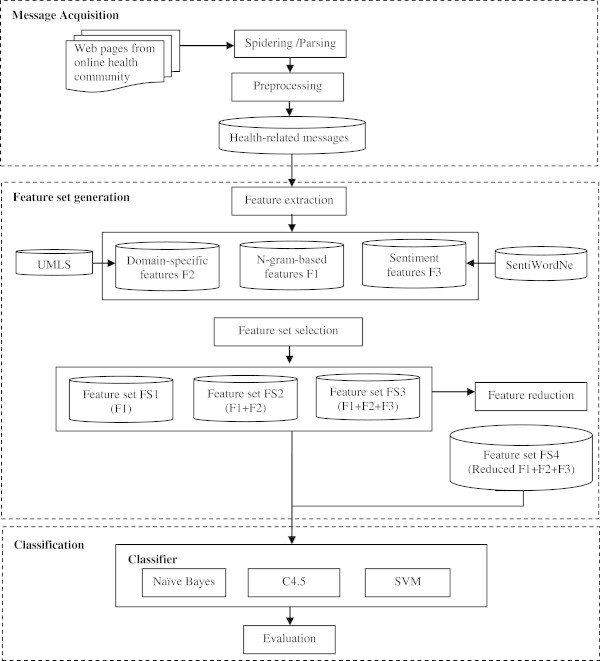
**The design framework of automatic topic identification.**

In this study, we proposed a topic identification method for identifying topic category of the messages in online health community. Our research framework consists of three steps: message acquisition, feature set generation, and classification, as shown in Figure 1.

### Message acquisition

In the data collection step, we first focused on an online health community and collected all the web pages by using the Web crawler software Offline Explorer. Then we parsed the pages to extract available health-related messages, meanwhile tagged these messages with their given topic category. Next, some noisy and unreliable data are filtered by text preprocessing, including stop words removal and word stemming.

### Feature set generation

In previous feature-based text classification studies, the vector space model was widely used for text representation (Salton et al. 1975). In traditional VSM, text is treated as an unordered bag-of-words (BOW) and each dimension of this space corresponds to a given word. However, text representation based on BOW model exists some apparent limitations. First, text space is generally of high dimensionality and text classification in such a high dimensional space is often computationally infeasible due to the curse of dimensionality. Then, many dimensions corresponding to rare words could mislead the classification model and cause the reduction of both classification accuracy and efficiency. To address this issue, we presented a novel feature selection method to find the significant features that represent the messages. In our study, three types of features were extracted: n-gram-based features, domain-specific features and sentiment features. These features have been adopted in previous text classification studies and proved effective for online text classification.

(1) N-gram-based features. As mentioned above, current text classification techniques are predominantly based on BOW model, that is, identifying terms with all the words occurring in the document and performing classification based mainly on the presence or absence of these words. Some researchers have used different classification techniques but the results demonstrated that various classification techniques produced similar results for BOW-based features. After that, some researchers attempted to improve the BOW-based text representation and their research results demonstrated that the addition of word n-grams (sequences of words of length n) to the BOW-based text representation indeed improved performance. However, sequences of length n > 3 were shown to be not useful and might decrease the performance. So in this study, n-gram-based features(n<=3) were incorporated into feature set.

(2) Domain-specific features. Studies have found that integrating domain-specific knowledge into textual feature representation could improve classification performance (Zhu et al. 2009). Since health-related messages in online community contain much medical knowledge, incorporating the medical domain-specific features could enhance the performance of topic identification significantly. So medical domain-specific features were introduced in this study as an additional feature dimension. In some previous studies, UMLS Metathesaurus as the world's largest repository of biomedical concepts, were widely used to extract medical terminology from medical text. So we also used UMLS to extract health-related medical terminology as domain-specific features. UMLS Metathesaurus consists of 1.7 million biomedical concepts where each concept is assigned to at least one of the 134 semantic types and the health-related semantic types used in our study were listed in Table 1.

**Table 1:** **The UMLS semantic types used**

Abbr.	Semantic types	Abbr.	Semantic types
Aapp	Amino acid, peptide, or protein	Imft	Immunologic factor
Acab	Acquired abnormality	Inpo	Injury or poisoning
Anab	Anatomical abnormality	lbpr	Laboratory procedure
Bdsy	Body system	Mobd	Mental or behavioral dysfunction
Blor	Body location or region	Neop	Neoplastic process
Bmod	Biomedical occupation or discipline	Orch	Organic chemial
Bpoc	Body part, organ, or organ component	Patf	Pathologic function
Diap	Diagnostic procedure	Phsu	Pharmacologic substance
Dsyn	Disease or syndrome	Sosy	Sign or symptom
Horm	Hormone	Topp	Therapeutic or preventive procedure

(3) Sentiment features. In addition to obtaining healthcare knowledge and medical information regarding disease-related symptoms, medication and treatments, patients involved in health online community also expect to obtain emotional support from other community members. Particularly for the patients diagnosed with cancers and chronic diseases, they usually posted some messages to vent their frustration, seek sympathetic encouragement and show compassion or empathy for others. So these messages usually contained many sentiment words with high sentiment polarity and these sentiment words could be used to measure effectively whether health-related messages provide informative support or emotional support, thus helpful to improve topic identification performance. In order to get these sentiment features, we exploited SentiWordNet as lexical resource to extract the terms with high sentiment polarity scores as sentiment features. SentiWordNet provides for each synset of WordNet a triple of polarity scores (positivity, negativity and objectivity) and now SentiWordNet consists of around 207,000 word-sense pairs or 117,660 synsets, which has been widely used as lexicon in recent sentiment analysis studies. So in this study we selected the terms with subjectivity score more than 0.5 as sentiment features and incorporated them into feature set.

Based on these three types of features, we built three feature sets in an incremental way: feature set FS1(F1), feature set FS2(F1+F2) and feature set FS3(F1+F2+F3). Features set FS1 was used as the baseline feature set to assess the performance of the other proposed feature sets. In addition, we adopted feature reduction techniques to remove irrelevant or redundant n-gram-based features and then built the fourth feature sets FS4(reduced F1+F2+F3). There are many feature reduction methods used in text classification studies where information gain (IG) is one of the most effective methods. So IG was adopted in our study to perform feature reduction. All features with IG greater than 0.0025 were selected (Abbasi et al. 2008). Let  denote the set of topic categories and term t denote a key n-gram-based feature. The information gain of term t is defined to be:1

where G(t) denote the information gain for feature t,P(c_i_) denote the probability of class c_i_,P(c_i_|t)denote the conditional probability of c_i_ given t, P(t) denote the probability of feature t occurring, and  denote the probability of feature t not occurring.

### Classification and evaluation

In this study, three state-of-the-art classification techniques, SVM with polynomial kernel, C4.5 and Naïve Bayes, were used to perform classification task. SVM is a powerful statistical machine-learning technique first introduced by Vapnik (Vapnik 1995). Due to the ability to handle millions of inputs and good performance, SVM was widely used in text classification studies. C4.5 is a decision-tree building algorithm developed by Quinlan (Quinlan 1986). Based on a divide-and-conquer strategy and the entropy measure, C4.5 focuses on classifying mixed objects into categories according to attribute values of objects. Based on Bayes’ theorem with strong independence assumptions, the Naïve Bayes classifier is a probabilistic classifier and uses the feature values of a new instance to estimate the probability of each category. It has also been used to perform text classification tasks in previous studies.

To assess the classification performance, we adopted the following standard metrics, accuracy, precision, recall and F-measure. These metrics have been widely used in text classification studies.2345

## Experiment

### Research testbed and data collection

In this study, we conducted our experiment on a popular breast cancer online community (http://apps.komen.org/Forums), supported by Susan G. Komen Breast Cancer Foundation. Breast cancer in the US continued to be a serious problem for women, characterized by high incidence and mortality rates and the detrimental impact on the quality of life. Many studies have shown that breast cancer was one of the most common cancers that Internet users searching for information online about (Castleton et al. 2011). The Susan G. komen forum is one of the largest online communities for breast cancer survivors and activists, and more than twenty thousand members got involved in it. More importantly, this online health community was already classified into several message boards based on different topics, thus providing a gold standard to evaluate the performance of our topic classification.

We collected the health-related messages in three message boards: treatment board, emotional support board and survivorship board. In the treatment board, members share their experiences, thoughts and advice on surgeries, reconstruction and chemotherapy. In the emotional support board, members post their well wishes and provide emotional support to those suffering from breast cancer. In the survivorship board, members share their experiences and advice on how they deal with life's daily challenges, and how to find a new routine of life while dealing with breast cancer. To evaluate the performance of our topic classification model, we randomly selected 4,041 messages from the collected data and tagged them with a predefined topic category label. Among these messages, 1,224 messages were tagged as treatment, 991 messages were tagged as emotional support, and 1,826 messages were tagged as survivorship.

## Results and discussion

**Figure 2:**
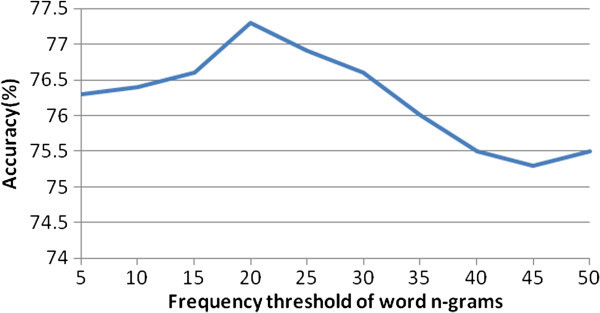
**Accuracies for different frequency thresholds for word n**-**grams.**

Topic classification in our study was performed using the Weka software package (Witten & Frank 2005), a popular suite of machine learning software written in Java and developed at the University of Waikato. Evaluation was done via a tenfold cross validation. In each fold, 90% of the data was used as training set and the remaining 10% of the data as test set. We first used baseline feature set FS1, that is n-gram-based features, to perform classification task. N-grams features used in our study consisted of unigrams, bigrams and trigrams with high frequency. Figure 2 showed the classification accuracy on different frequency thresholds for word n-grams. We could see that the accuracy increased first and then decreased as the frequency threshold increased. We could get the highest accuracy when frequency threshold was set as 20.

### Comparison of different feature sets

**Figure 3:**
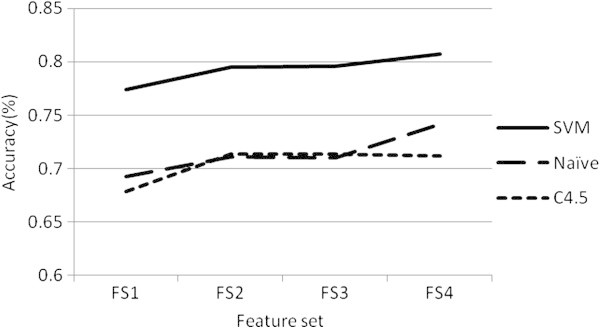
**Accuracies using different feature sets and classification techniques.**

We examined the classification performance using different feature sets and classification techniques. From Figure 3 we could see intuitively that the highest accuracy was achieved when using feature set FS4 and SVM classifier. In terms of feature sets, FS2 outperformed FS1 consistently when using any of the three classification techniques. However, compared to feature set FS2, there seemed to be no significant performance improvement when using feature set FS3 and FS4.

To further examine the performance of different feature sets, we performed pairwise t-tests on accuracy and F-measure. The t-tests were conducted by randomly taking sample data and then performing tenfold cross validation. We repeated the process 10 times and then got the t-test results, shown in Table 2. The results demonstrated that the classification accuracy and F-measure were significantly higher when using feature set FS2 than using feature set FS1(p-value<0.01 on both accuracy and F-measure when FS1<FS2), indicating that adding domain-specific features improved the topic classification performance significantly. However, in the comparison of feature set FS2 and FS3, FS3 outperformed FS2 significantly when using SVM classifier, while performance difference between FS3 and FS2 was not significant when using C4.5 and Naïve Bayes classifiers, indicating that adding sentiment features may not be effective to distinguish the messages with different topics when using C4.5 and Naïve Bayes classifiers. In the comparison of feature set FS3 and FS4, we can see that FS4 outperformed FS3 significantly when using Naïve Bayes classifier and SVM classifier, indicating that using feature reduction could improve the topic classification performance significantly.

**Table 2:** **Pairwise t**-**tests on accuracy and F**-**measure for different feature sets**

Feature set	P-value on accuracy			P-value on F-measure		
	C4.5	Naïve Bayes	SVM	C4.5	Naïve Bayes	SVM
FS1<FS2	<0.0001	<0.0001	<0.0001	<0.0001	<0.0001	0.0002
FS2<FS3	0.1717	0.0583	0.0036	0.3392	0.2104	0.0075
FS3<FS4	0.0282	<0.0001	<0.0001	0.0425	<0.0001	<0.0001

### Comparison of different classification techniques

From Figure 3 we could see that, in terms of classification techniques, SVM consistently outperformed C4.5 and Naïve Bayes when using any of the four feature sets. We further conducted pairwise t-tests on accuracy and F-measure using different classification techniques. The processes are very similar to those in the comparison of different feature sets. From Table 3 we can see that the performance differences between C4.5 and Naïve Bayes were statistically insignificant. However, SVM achieved significantly higher accuracy and F-measure than C4.5 and Naïve Bayes when using any of the four feature sets, indicating that SVM is the best classification technique used in our topic classification model.

**Table 3:** **Pairwise t**-**tests on accuracy and F**-**measure for different classification techniques**

Classifiers	P-value on accuracy				P-value on F-measure			
	FS1	FS2	FS3	FS4	FS1	FS2	FS3	FS4
C4.5<Naive	0.2997	0.3439	0.3667	0.00146	0.03679	0.1158	0.0748	<0.0001
C4.5<SVM	<0.0001	<0.0001	<0.0001	<0.0001	<0.0001	<0.0001	<0.0001	<0.0001
Naive<SVM	<0.0001	<0.0001	<0.0001	<0.0001	<0.0001	<0.0001	<0.0001	<0.0001

### Comparison of different topics

We made a further analysis of the difference between the three topic groups. We examined the precision, recall and F-measure of different topic groups using SVM classifier. From Table 4 we can see that the highest precision was obtained in emotional support group. One possible reason is that the messages in emotional support topic group contain many sentiment words and few medical terminologies, which makes it easier to distinguish these emotional support messages from the other two types of messages using our feature-based classification method. Oppositely, survivorship topic group achieved the best recall but lower precision. The reason may be that many messages in survivorship topic group contain many medical terminology and sentiment words, and these domain-specific features and sentiment features could not be used to distinguish the topics of these messages, which makes many irrelevant messages misclassified into survivorship group. Overall, however, we could get 81.2% precision, 80.7% recall and 80.6% F-measure on average when using feature set FS4, indicating that our topic classification model was effective for identifying health-related topics of the messages in online health community.

**Table 4:** **Performance measures of different topic groups**

Feature set	Topic	Precision	Recall	F-measure
FS1 (F1)	Treatment	75.2%	70.3%	72.6%
	Emotional	82.2%	72.4%	77.0%
	Survivorship	76.2%	87.1%	81.3%
	Average	77.6%	77.4%	77.2%
FS2 (F1+F2)	Treatment	77.0%	74.1%	75.5%
	Emotional	84.5%	75.3%	79.7%
	Survivorship	78.4%	87.2%	82.6%
	Average	79.7%	79.5%	79.4%
FS3 (F1+F2+F3)	Treatment	77.2%	74.0%	75.6%
	Emotional	84.6%	75.3%	79.7%
	Survivorship	78.3%	87.5%	82.6%
	Average	79.8%	79.6%	79.5%
FS4 (selected F1+F2+F3)	Treatment	80.2%	73.9%	76.9%
	Emotional	87.9%	75.5%	81.2%
	Survivorship	77.2%	90.3%	83.2%
	Average	81.2%	80.7%	80.6%

## Conclusions and future research

With the development of online health community, automatic topic identification of health-related messages could assist patients in searching their interesting topics and facilitate their involvement in online health community. So in this paper, we proposed a topic identification model based on text classification techniques. To evaluate the effectiveness of this model, we conducted experiments on one breast cancer online community using different feature sets and classification techniques. We found that n-gram-based features, domain-specific features and sentiment features have significant influence on improving topic identification performance. In terms of classifiers, SVM outperformed C4.5 and Naïve Bayes significantly. So we finally found the by combining of feature set FS4 and SVM classifier, our topic classification model could produce the best classification results. The experimental results also demonstrated that the proposed approach could identify the topics of online health-related messages effectively.

The paper also has some limitations that need to be considered further. First, the study has proved that incorporating medical domain-specific features and sentiment features could enhance the performance of topic classification significantly, however, some other features should be considered to be used to further improve the performance. For example, messages within a single thread most likely belong to the same topics, so these structural features should be considered in the feature set in the further research. Second, we adopted information gain to remove irrelevant or redundant n-gram-based features to generate better feature sets, however, there are some other feature reduction methods such as Markov blanket, which were proved effective in some studies. So further research could explore and compare the performance of different feature reduction methods to obtain the best feature sets for topic classification. Lastly, other classification techniques, in addition to SVM, C4.5 and Naïve Bayes, should be considered to improve the performance of topic identification in further research.
